# Immunological Profile of Silent Brain Infarction and Lacunar Stroke

**DOI:** 10.1371/journal.pone.0068428

**Published:** 2013-07-09

**Authors:** Paola Sarchielli, Katiuscia Nardi, Davide Chiasserini, Paolo Eusebi, Michela Tantucci, Vittorio Di Piero, Marta Altieri, Carmine Marini, Tommasina Russo, Mauro Silvestrini, Isabella Paolino, Paolo Calabresi, Lucilla Parnetti

**Affiliations:** 1 Neurologic Clinic, Department of Medical and Surgical Specialties and Public Health, University of Perugia, Ospedale Santa Maria della Misericordia, Perugia, Italy; 2 Department of Epidemiology, Regional Health Authority of Umbria, Perugia, Italy; 3 Department of Neurological Sciences, Sapienza University of Rome, Rome, Italy; 4 Department of Internal Medicine and Public Health, University of L’Aquila, L’Aquila, Italy; 5 Neurologic Clinic, Neuroscience Department, Marche Polytechnic University, Ospedali Riuniti Ancona, Ancona, Italy; 6 Fondazione S. Lucia - Istituto di Ricovero e Cura a Carattere Scientifico (I.R.C.C.S.), Rome, Italy; University of Muenster, Germany

## Abstract

Neuroinflammation is believed to be involved in the pathophysiological mechanisms of silent brain infarcts (SBI). However, the immunological profile of SBI has been scarcely investigated. In the context of a national research project named SILENCE, aimed at investigating clinical, biochemical and pathogenic features of SBI, we have measured the plasma profile of some inflammatory-related molecules in SBI patients (n = 21), patients with recent lacunar infarcts (LI, n = 28) and healthy controls (n = 31), consecutively enrolled in four Italian centres. A panel of chemokines (MIG, CTACK, IL16, SDF1a, MCP1), growth factors (SCF, SCGFb, HGF, IL3), immunoglobulin-type adhesion molecules (ICAM1, VCAM1), proinflammatory cytokines (IL18, INFa2, MIF, IL12p40), cell surface receptors on T-cells (IL2Ra), and inductors of apoptosis (TRAIL) was assessed in plasma samples by Luminex xMAP™ technology. Immunological parameters were compared using non-parametric statistics and performance to distinguish SBI and LI was evaluated by receiver operating characteristic (ROC) analysis. Plasma levels of ICAM1 were significantly higher in both SBI and LI patients as compared to controls (SBI≥LI>Ctrl). A different trend was observed for IL16 (SBI<LI>Ctrl), SCF (LI<SBI>Ctrl) and SCGFb (SBI>LI<Ctrl). SBI subjects had significantly increased levels of MIG when compared to controls (LI≤SBI>Ctrl) and IL18 when compared to LI patients (Ctrl≤SBI>LI). All the other immunological markers did not significantly differ among groups. According to ROC analysis, the best predictor for SBI condition was the chemokine MIG (AUC = 0.84, sensitivity 86%, specificity 77%), while SCF had the best performance in distinguishing LI patients (AUC = 0.84, sensitivity 86%, specificity 68%). These results confirm the involvement of inflammatory processes in cerebrovascular disorders, particularly in SBI, a very common age-related condition. The differences in plasma profile of inflammatory molecules may underlie different pathological mechanisms in SBI and LI patients.

## Introduction

The term silent brain infarction (SBI) refers to cerebral infarcts detected by brain imaging in subjects without any related clinical manifestation [1]. SBI are radiologically similar to lacunar infarcts (LI). Variations of magnetic resonance imaging (MRI) characteristics and diagnostic criteria for MRI-defined SBI may lead to great discrepancies in the definition of SBI [1], [2]. In about half of published studies, SBI was defined as hypointense area on T1 and hyperintense on T2-weighted images sized ≥3 mm [1].

Although SBI are commonly found in the elderly, they can be detected in people of any age with a higher prevalence than LI [2]. SBI have been considered as benign unspecific age-dependent findings, but recent studies show that they are consistently associated with (i) increased risk for stroke independently from co-occurrence of vascular risk factors [2]–[7] (ii) cognitive decline, including a higher rate of conversion from mild cognitive impairment to dementia [2], [4], [5] (iii) depression [8] and (iv) disability [2].

SBI and LI are believed to share similar pathogenic mechanisms - they may be of cardio-embolic origin, or caused by atherosclerotic processes taking place mostly in the small vessel walls [2], [3] and vascular risk factors, such as advanced age, hypertension, metabolic syndrome, coronary artery disease [2], [6].

Inflammatory mechanisms have been repeatedly invoked in the pathogenesis of cerebrovascular diseases, namely acute stroke and SBI. With respect to this latter condition, only in few reports the potential role of inflammation has been investigated, and higher levels of interleukin-6 and C-reactive protein have been shown [9], [10].

In this study, we aimed at investigating whether plasma levels of molecules expressing the inflammatory state – i.e., cytokines, chemokines, adhesion molecules, cell surface receptors, inductors of apoptosis and transforming growth factors - may show a differential pattern in SBI and LI patients.

## Methods

### Ethics Statement

The study has been carried out in accordance with The Code of Ethics of the World Medical Association (Declaration of Helsinki) for experiments involving human subjects. The SILENCE study protocol was approved by the institutional Ethics Committees of all participating centres (supporting information, table S1). Written informed consents were obtained from all subjects prior to the entry into the study.

### Study Design and Subjects

Our investigation is an ancillary study of the national research project named SILENCE, involving 10 Italian centres and aimed at investigating neuropsychological, neuroradiological, neurosonographic, and immunological features of clinically asymptomatic subjects showing SBI at MRI. Our centre was responsible for the determination of plasma levels of several circulating immunological parameters expressing the inflammatory state. Plasma samples suitable for such determinations were obtained from four Centres (Perugia, Rome, Ancona, L’Aquila). Accordingly, 21 SBI subjects, 28 LI patients and 31 healthy subjects were recruited.

### Inclusion/Exclusion Criteria

Inclusion criteria for SBI patients were: (i) male/female aged ≥45 years, (ii) negative history of stroke and/or transient ischemic attack, (iii) MRI positive for SBI (see below), (iv) normal neurological evaluation, (v) absence of any sign of cognitive impairment (Mini Mental State Exam score ≥28) and (vi) Eligibility criteria for LI patients were: (i) male/female aged ≥45 years, (ii) diagnosis of first-ever LI, defined according to the Oxfordshire Community Stroke Project criteria [11], occurred from 4 to 6 weeks before enrolment plus a corresponding lacunar lesion on MRI, (iii) absence of post-stroke complications and any sign of cognitive impairment. Neurologically healthy subjects of both sexes, aged ≥30 years, showing normal brain MRI (i.e., no SBI) were enrolled as control group. None of the SBI and control subjects included in the study has been taken any anti-inflammatory drug for at least one week before plasma collection. All LI patients were taking clopidogrel 75 mg daily. None of patient and controls had history of recent infections (acute or chronic) and immunological diseases. None of them was febrile at the time of blood sampling. Urine analysis was normal, no changes were found in the blood count and the reactive C protein (RCP) was in the normal range. No evidence of pulmonary infection at the torax x ray was found in the subjects enrolled.

### Vascular Risk Factor Assessment

All major vascular risk factors were systematically assessed. We defined (i) hypertension as a history of elevated blood pressure (≥140 mmHg in systolic value and/or ≥90 mmHg in diastolic value in more than 3 measurements) requiring antihypertensive therapy, (ii) diabetes mellitus in presence of fasting plasma glucose exceeding 126 mg/dL or 200 mg/dL 2 hours after meal; (iii) hypercholesterolemia as a total plasma cholesterol >200 mg/dL or Low Density Lipoprotein cholesterol >170 mg/dL in the previous 12 months, smoking habit, obesity (body mass index ≥25), and carotid disease (carotid stenosis >50%).

### MRI-protocol for SBI and LI Definition

All the MRI exams were carried out by the same expert trained neuroradiologist. The evaluators were masked for the respective study groups. Participants underwent a brain MRI 1.5 Tesla following a standardized procedure. Patients were positioned in comfortable way to avoid even minimal movements of the head. A scout in the three space plans was performed, positioning the sagittal scans on the median line, for a better visualization of the corpus callosum. Scans have explored the whole brain; they have been positioned with the axial plan parallel to the lower margin of the corpus callosum.

The following sequences were performed: diffusion weighted imaging (DWI), proton density-T2: TR 2000–4500, TE 15–50/80–120; f-FLAIR: 1–2, TR 7000–11000, TE 150/200, TI 1500–2000, Echo-train-length 30–50; Gradient-echo (FFE-FLASH): TR 600–800, TE 20–30, Flip angle 15–25; and three-dimensional-T1 (SPGR or MPRAGE): TR 20–30, TE 5–10, Flip angle 50. For all the scans, the same number of slices has been obtained with the following parameters: 44–48 slices of the thickness of 3 mm (gap: 0 mm), FOV 25 cm, matrix 256 xes 256, L/R coding phase.

SBI were identified in agreement with Vermeer et al. [2]: an infarction at MRI defined as a high signal area on the T2-weighted images of at least 3 mm in diameter. The scans in proton density were used to distinguish an infarct from a dilation of the perivascular spaces. Moreover, the lesions in the white matter should appear as an area of low signal on the T1-weighed images to distinguish them from the leukoaraiosis. LI was defined as a focal hyperintensity on T2-weighted images, hypointensity on T1, and positive diffusion-weighted findings in the acute phase of stroke [12].

To avoid a great variation in the lesional load of SBI patients, we decided to include patients with bilateral SBI with a minimum of 3 and a maximum of 8 lesions.

### Immunological/Inflammatory Profiling

Collection of samples from all participants was performed in the morning after overnight fasting. Blood (approximately 5 ml) was obtained by forearm vein puncture and drawn in ethylenediaminetetraacetic acid (EDTA) coated tubes. Samples were centrifuged at 3000×g for 10 min and the resulting plasma was aliquoted into polypropylene tubes, which were immediately frozen at −80°C, pending analyses. All plasma samples were analyzed by multiplex immunoassay based Luminex xMAP™ technology using a multiplex kit. Multiplex kits and reagents were purchased from Biorad (Hercules CA, USA).

Twenty-four analytes were quantified: cutaneous T-cell-attracting chemokine (CTACK), growth regulated oncogene-alpha (GROa), hepatocyte growth factor (HGF), intercellular adhesion molecule-1 (ICAM1), interferon alpha-2 (INFα2), interleukin-1a (IL1a), interleukin-2 receptor-alpha (IL2Ra), interleukin-3 (IL3), interleukin-12 p40 (IL12p40), interleukin-16 (IL16), interleukin-18 (IL18), leukocyte inhibitory factor (LIF), monocyte chemoattractant protein-3 (MCP3), monocyte colony stimulating factor (MCSF), macrophage migration inhibitory factor (MIF), monocyte chemoattractant protein-1 (MCP1), monokine induced by gamma-interferon (MIG), nerve growth factor-beta (NGFb), stem cell factor (SCF), stem cell growth factor-b (SCGFb), stromal cell-derived factor-1a (SDF1a), tumor necrosis factor-α-related apoptosis-inducing ligand (TRAIL), tumor necrosis factor-beta (TNFb), vascular cell adhesion molecule-1 (VCAM1).

All measurements were performed at least in duplicate, following the instructions from the manufacturer. Briefly, plasma samples were thawed on ice and diluted 1∶4 with sample diluent. Samples were dispensed on the microtiter plate and incubated for 30 minutes at room temperature with beads conjugated with the specific monoclonal antibodies for the different analytes. Biotinylated secondary antibody was then added and incubated with the beads at room temperature for 30 minutes. Streptavidin conjugated phycoerytrin was added to all wells and incubated for 10 minutes at room temperature. After plate washing, samples were quantified using Luminex 200 instrument (BIORAD Hercules CA, USA). All the determinations were done in duplicate. Standard curves were built with pre-mixed standards included in the kits. Median fluorescence of the samples was interpolated using five parameter logistic fit method embedded into the software Bioplex Manager v. 5.0 (BIORAD Hercules CA, USA).

### Statistical Analysis

The statistical analysis was carried out using Statistical Analysis Software version 9.1. Median values and interquartile ranges (IQR) for continuous variables, percentages for categorical ones were calculated.

Demographic variables and vascular risk factors were compared among the three groups, using chi-square statistics for categorical variables and Kruskal-Wallis statistics for continuous variables. Cytokine levels were compared among groups using non parametric ANOVA adjusting for age, gender and vascular risk factors (hypertension, diabetes mellitus, atrial fibrillation and carotid atheroma, hyperlipemia). The data were adjusted for co-medications considered as drug classes (antihypertensives, antidiabetics, statins, and anti-arrhythmic agents). Post-hoc paired comparisons were calculated according to Bonferroni correction. For all tests, a probability value <0.05 was considered as statistically significant.

Finally, we tested the cytokines involved in SBI or LI by estimating their discriminative power by means of receiver operating characteristic (ROC) analysis. ROC curves were built only for cytokines which showed significant difference after post-hoc analysis, healthy subjects were considered as reference group. Cut-off values were chosen as the point of the curve with the highest sum of specificity and sensitivity. Area Under the Curve (AUC) with its 95% confidence interval (CI) were calculated. For AUC, the following ranges were considered: 0.70>AUC>0.80 indicating acceptable discrimination and AUC >0.80 good discrimination [13].

## Results

### Characteristics of the Study Population

Demographic details of the subjects studied are reported in Table 1. There was no difference in sex distribution among the three groups studied. SBI subjects were younger than LI patients, and both groups were older than controls. Healthy subjects did not show major vascular risk factors; only hypercholesterolemia was observed in a few cases. Carotid disease was more frequently found in LI patients than in SBI and control subjects.

**Table 1:** Characteristics of study population at baseline.

Demographics and risk factors	SBI (n = 28)	LI (n = 21)	Controls (n = 31)	p-value
Age, years	64 (53–70)	71 (67–73)	36 (28–50)	**<0.0001**
Female gender	22 (78.6)	11 (52.4)	20 (64.5)	0.0962
Hypertension	16 (57.1)	18 (85.7)	1 (3.2)	**<0.0001**
Diabetes mellitus	2 (7.1)	9 (42.9)	0 (0.0)	**<0.0001**
Hypercholesterolemia	9 (32.1)	9 (42.9)	3 (9.7)	**0.0183**
Atrial fibrillation	0 (0.0)	6 (28.6)	0 (0.0)	**0.0001**
Current smoking	4 (14.3)	4 (19.0)	0 (0.0)	0.0503
Smoking habit	9 (32.1)	4 (19.0)	1 (3.2)	**0.0111**
Carotid atheroma	2 (7.1)	12 (57.1)	0 (0.0)	**<0.0001**
Body Max Index ≥25	12 (42.9)	4 (19.0)	3 (9.7)	**0.0069**

Age is given as median (interquartile range). Gender and vascular risk factors are reported as absolute number of subjects (%). P-values are calculated using chi-square statistics (categorical variables) and Kruskal-Wallis statistics (continuous variables).

SBI: silent brain infarcts; LI: lacunar stroke.

### Immunological/Inflammatory Profiling of the Study Population

Seventeen molecules out of the 24 measured in plasma were included in the analysis. The levels of 7 molecules (IL-1a, GROa, LIF, MCP3, MCSF, bNGF, TNFb) were constantly below the detection limit. Table 2 shows the median (IQR) levels of detectable cytokines, chemokines, and inflammatory molecules statistical significance after adjustment for baseline. Levels of CTACK, HGF, IL2Ra, IL3, IL16, IL18, IFNa2, MIG, ICAM1, SCF, SCGFb, TRAIL, SDF1a and VCAM1 significantly differed among groups, whereas IL12p40, MIF and MCP1 levels did not.

**Table 2:** Comparison of immunological/inflammatory profiling between SBI, LI and controls.

Biomarker	SBI	LI	Controls	p-value
**CTACK**	1270 (919–1470)	855 (593–1146)	834 (709–1210)	**0.0012**
**HGF**	199 (161–279)	242 (180–363)	149 (121–180)	**0.0005**
**IL2Ra**	105 (60–146)	85 (58–112)	67 (49–90)	**0.0093**
**IL3**	26 (15–29)	17 (7–27)	12 (5–22)	**0.0463**
**IL12p40**	122 (61–167)	68 (61–182)	88 (16–122)	0.0864
**IL16**	149†(108–270)	270‡ (262–381)	143 (66–312)	**0.0172**
**IL18**	74†(54–92)	36 (22–62)	59 (36–76)	**0.0029**
**IFNa2**	45 (35–49)	56 (52–68)	40 (29–52)	**0.0024**
**MIF**	380 (214–604)	375 (281–684)	453 (316–660)	0.3699
**MIG**	1412*(843–1964)	911 (613–1328)	528 (393–711)	**<0.0001**
**MCP1**	29 (22–41)	29 (23–35)	32 (25–37)	0.7766
**ICAM1**	65063*(51761–82522)	61353‡(50221–68068)	45751 (38723–55532)	**<0.0001**
**SCF**	156* ^.^ †(123–183)	67 (40–81)	110 (77–138)	**<0.0001**
**SCGFb**	38442†(30952–48524)	20914‡ (13675–26198)	33575 (27320–47087)	**0.0040**
**SDF1a**	139 (117–203)	281 (240–361)	159 (90–240)	**0.0155**
**TRAIL**	72 (64–89)	39 (31–58)	34 (29–67)	**0.0028**
**VCAM1**	150483 (135789–183869)	175767 (152801–184346)	130669 (114984–154163)	**0.0442**

Data are given as median value (interquartile range) of cytokines blood levels (pg/mL).

P-values are reported for non-parametric ANOVA (adjusted for gender, age, vascular risk factors and co-medications considered as classes - antihypertensives, antidiabetics, statins, and anti-arrhythmic agents-). For significant overall group effect, p values of Bonferroni adjusted multiple comparisons are reported.

* p<0.05 SBI vs. Controls;

† p<0.05 SBI vs. LI;

‡ p<0.05 LI vs. Controls.

CTACK: cutaneous T-cell-attracting chemokine; HGF: hepatocyte growth factor; ICAM1: intercellular adhesion molecule-1; IL12p40: interleukin-12 p40; IL-16: interleukin-16; IL18: interleukin-18; IL2Ra: interleukin-2 receptor-alpha; IL3: interleukin-3; INFα2: interferon alpha-2; LI: patients with lacunar stroke; MCP1: monocyte chemoattractant protein-1; MIF: macrophage migration inhibitory factor; MIG: monokine induced by gamma-interferon; SBI: patients with silent brain infarcts; SCF: stem cell factor; SCGFb: stem cell growth factor-b; SDF1a: stromal cell-derived factor-1a; TRAIL: tumor necrosis factor-α-related apoptosis-inducing ligand; VCAM1: vascular cell adhesion molecule-1.

### Post-hoc Pairwise Analysis

**Figure 1:**
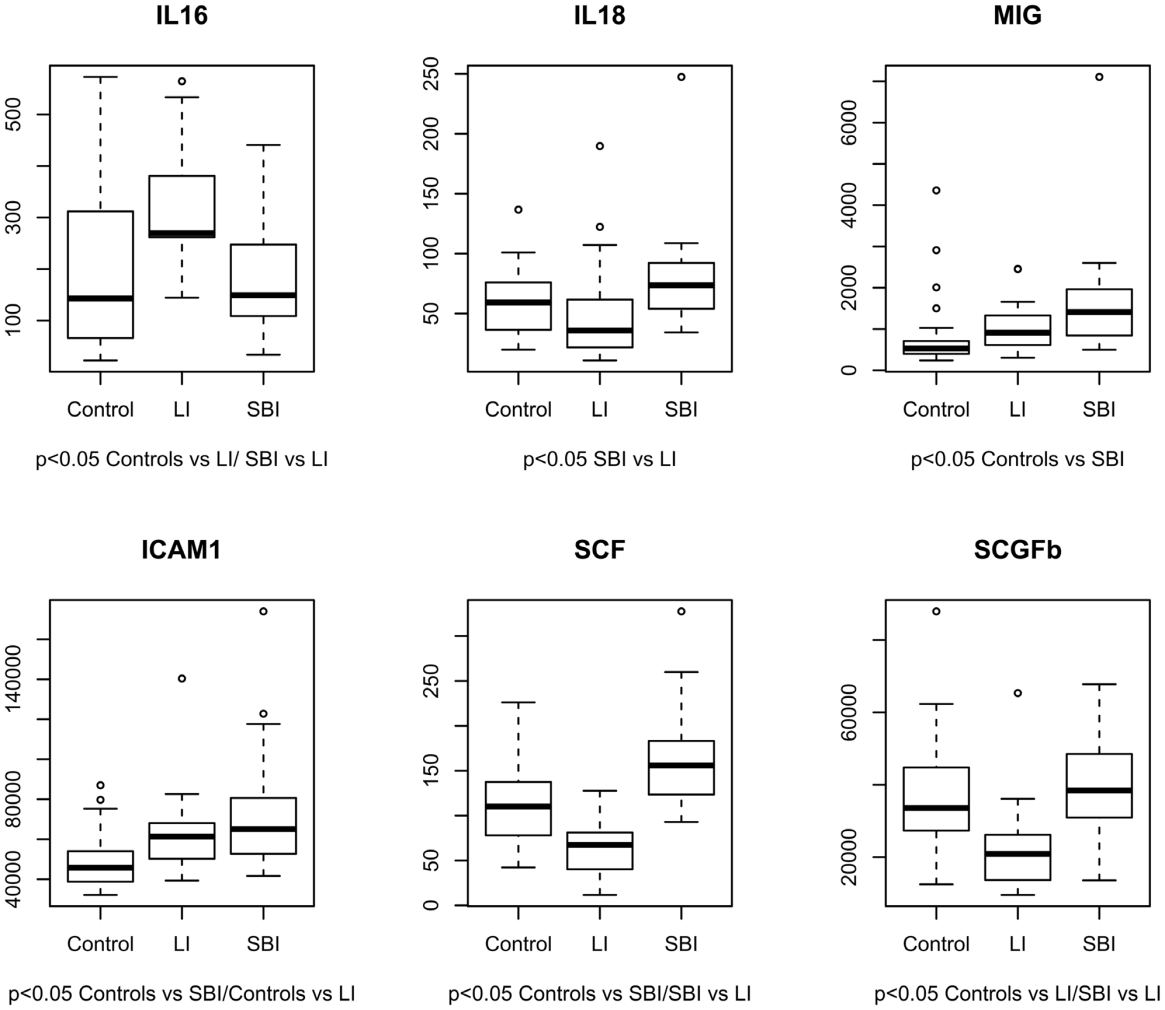
Post-hoc pairwise comparisons of immunological/inflammatory profiling between SBI, LI and healthy control subjects. Abbreviations: ICAM1: intercellular adhesion molecule-1; IL-16: interleukin-16; IL18: interleukin-18; MIG: monokine induced by gamma-interferon; SCF: stem cell factor; SCGFb: stem cell growth factor-b. LI: lacunar stroke; SBI: silent brain infarcts.

Levels of ICAM1 were significantly higher in both SBI and LI patients as compared to controls, without significant difference between two groups (SBI≥LI>Ctrl) (Table 2 and Figure 1).

Furthermore, IL-16 levels were significantly increased in Li compared to SBI and controls, conversely SCF and SCFGb levels were significantly decreased in LI compared both SBI and control groups (Table 2 and Figure 1).

SBI subjects had significantly increased levels of MIG when compared to controls (LI≤SBI>Ctrl), and IL18 when compared to LI patients (Ctrl≤SBI>LI). All the other immunological markers did not significantly differ among groups.

### Discriminating Cytokines on SBI and LI Compared to Controls

**Figure 2:**
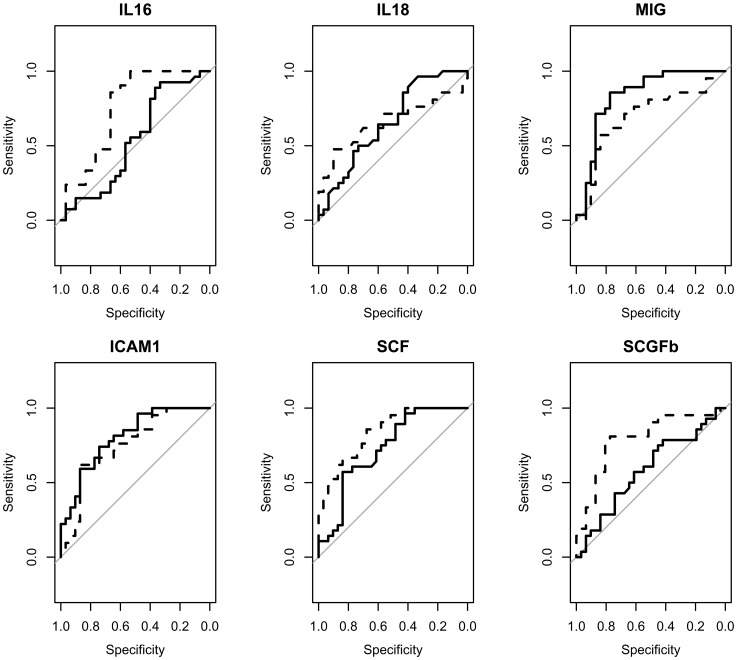
ROC curves in SBI (continuous lines) and LI patients (broken lines) of each analyte, showing a statistically significant difference in the comparison among SBI, LI and Controls. Abbreviations: ICAM1: intercellular adhesion molecule-1; IL-16: interleukin-16; IL18: interleukin-18; MIG: monokine induced by gamma-interferon; SCF: stem cell factor; SCGFb: stem cell growth factor-b. LI: lacunar stroke; SBI: silent brain infarcts.

Figure 2 illustrates the ROC curves in SBI and LI patients of each analyte, showing a statistically significant difference in the comparison among SB, LI and healthy group. Predictive values, AUC (95% CI), sensitivity and specificity of the same analytes in SBI and LI patients are reported in Table 3.

**Table 3:** Predictive values (pg/mL), AUC (95% CI), sensitivity and specificity of each analyte.

	LI				SBI			
Biomarker	Cut-off	AUC (95%CI)	Sensitivity	Specificity	Cut-off	AUC (95%CI)	Sensitivity	Specificity
**ICAM1**	≥58250	0.75 (0.62–0.89)	0.62	0.87	≥53699	0.81 (0.70–0.92)	0.74	0.74
**IL16**	≥239	0.75 (0.62–0.89)	0.99	0.53	≥78	0.54 (0.38–0.69)	0.93	0.33
**IL18**	≤26	0.66 (0.50–0.82)	0.47	0.90	≥75	0.66 (0.52–0.80)	0.50	0.73
**MIG**	≥908	0.69 (0.54–0.85)	0.57	0.84	≥740	0.84 (0.73–0.94)	0.86	0.77
**SCF**	≤84	0.84 (0.74–0.95)	0.86	0.68	≥150	0.73 (0.60–0.86)	0.61	0.77
**SCGFb**	≤26993	0.79 (0.66–0.92)	0.81	0.77	≥31523	0.58 (0.44–0.73)	0.71	0.48

Statistical significance for AUC is underlined.

ICAM1: intercellular adhesion molecule-1; IL-16: interleukin-16; IL18: interleukin-18; MIG: monokine induced by gamma-interferon; SCF: stem cell factor; SCGFb: stem cell growth factor-b. LI: lacunar stroke; SBI: silent brain infarcts.

Increased levels of ICAM1 were predictive for both SBI and LI condition, but ICAM1 had a more good discrimination for SBI patients. LI subjects had lower median plasma levels of ICAM1 and higher cut-off point for ICAM1 together with a lower discriminating AUC value in comparison to SBI patients, suggesting a lower discrimination for ICAM1 in LI than SBI subjects (Table 3 and Figure 2). Levels of SCF showed an opposite cut-off points as predictors for SBI (increased levels) and LI condition (lower levels) (Table 3 and Figure 2). Higher levels of MIG were predictive for SBI condition (AUC, 95% CI: 0.84, 0.73–0.95) while higher levels of IL16 (AUC, 95% CI: 0.75, 0.62–0.89) and lower levels of SCGFb (AUC, 95% CI: 0.79, 0.66–0.92) were discriminating for LI patients (Table 3 and Figure 2). Plasma levels of IL18 had poor discriminative power for both SBI and LI condition.

## Discussion

In the present study, we investigated the plasma levels of a wide range of cytokines, chemokines, inflammation-related molecules, cellular receptors, inductors of apoptosis and transforming growth factors, which are involved in the immunomodulatory/inflammatory mechanisms taking place in vascular diseases (Table 4). The aim was to verify their potential involvement in cerebrovascular disorders due to cerebral small vessel disease, namely SBI and LI. We found that ICAM1,IL16, IL18, MIG. SCF, SCGFb are altered and differentially expressed in SBI and LI patients. Higher plasma levels of ICAM1, MIG and SCF better predict SBI; in this condition also levels of IL18 and SCGFb have been found significantly higher. Increased ICAM1 and IL16 as well as lower levels of both SCF and SCGFb reliably discriminate LI from SBI.

**Table 4:** Immunomodulatory and inflammatory activity of the investigated molecules.

	Role in vascular pathophysiology	Evidence in vascular diseases
**Chemoattractant molecules**		
**CTACK**	Neuroinflammatory activity in transient cerebral ischemia [37].	Increase in acute coronary disease [23].
**IL16**	Increase of poststroke inflammatory response [22].	Early increase after focal cerebral infarction [23].
**MCP1**	Early induction after cerebral ischemia [38]. Increase of both poststroke inflammatory response and migration of neural stem cells [39]–[41]. Proatherosclerotic effects [42].	Predictor of ischemic stroke independently of vascular risk factors [43] and death or myocardial infarction in coronary syndromes [44].
**MIG**	Potent inhibitor of angiogenesis [27]. Proatherosclerotic effects [30].	Increase in acute coronary disease [23]. Predictor of cardiac event in acute coronary syndrome [28]. Inhibitor of extent of coronary collaterals in chronic heart disease [29].
**SDF1a**	Induction of endothelial progenitor cells chemotaxis [45].	In acute ischemic stroke, low levels are associated with large lesion volumes and worse outcomes [33].
**Growth factors**		
**IL3**	Promoter of neovascularization [46].	–
**HGF**	Long-term neuroprotective effects [47].	Increase in ischemic stroke patients, in subjects with vascular risk factors [48] and in diabetics with leukoaraiosis [49].
SCF*	Neuroprotective action on brain repair after stroke [16].	Positive correlation with circulating endothelial progenitor cells in acute ischemic stroke patients [33].
**SCGFb**	Hematopoietic progenitor cells [34].	Down-regulation in coronary collateral blood samples of patients with coronary occlusions [35].
**Immunoglobulin-type adhesion molecules**		
**ICAM1**	Overexpression in microvessels of cerebral ischemic zone,increased leukocyte adherence and activation [50].	Negative correlation with circulating endothelial progenitor cells in acute ischemic stroke. Predictor of tissue injury and stroke severity [21].
**VCAM1**	Progression of penumbral tissue in stroke [19].	Increase in cerebral infarcted areas [20].
**Proinflammatory cytokines**		
**IL12p40**	Worse outcome of brain injury after ischemia [51].	Polymorphisms are not associated to coronary disease severity [52] or myocardial infarction [53].
**IL18**	Proatherosclerotic effects [31].	Predictor of poor stroke outcome [24], post-stroke depression [54], cardiovascular death [26], coronary events [25], but not recurrent stroke [55].
**INFa2**	Worsening of ischemia-induced brain damage [56].	–
**MIF**	Promoter of neuronal death and severe neurologic deficits in stroke [57]. Proatherogenetic effects [58].	Up-regulation of gene expression after stroke induced by hypoxia [59].
**Cell surface receptor on T cell for its activation**		
**IL2Ra**	Suppression of neurogenesis after removal of ILR2a-T cells in stroke model [60].	Enhanced expression on T cell in stroke patients [61].
**Inductor of apoptosis**		
**TRAIL**	Expression in areas vulnerable to ischemia may underlie selective neuronal death after transient global ischemia [62].	–

CTACK: cutaneous T-cell-attracting chemokine; HGF: hepatocyte growth factor, ICAM1: intercellular adhesion molecule-1; IL12p40: interleukin-12 p40; IL-16: interleukin-16; IL18: interleukin-18; IL2Ra: interleukin-2 receptor-alpha; IL3: interleukin-3; INFα2: interferon alpha-2; MCP1: monocyte chemoattractant protein-1; MIF: macrophage migration inhibitory factor; MIG: monokine induced by gamma-interferon; SCF: stem cell factor; SCGFb: stem cell growth factor-b; SDF1a: stromal cell-derived factor-1a; TRAIL: tumor necrosis factor-α-related apoptosis-inducing ligand; VCAM1: vascular cell adhesion molecule-1.

In LI patients, the increased ICAM1 and IL16 plasma levels may be interpreted as the persistence of a proinflammatory state [14]. In our series LI patients were in a post-acute clinically stable phase and under pharmacological treatment with clopidogrel for secondary prevention. This anti-platelet agent has a minor influence on inflammatory markers when administered at high doses [7], [15]–[18]. Therefore, it can be assumed that its effect on inflammatory parameters measured in this investigation is negligible. Furthermore our patients received clopidogrel for gastric problems and not because of a repeated ischemic event (excluding LI) excluding therefore that the latter condition could be another explanation for increased levels of pro-inflammatory cytokines such as ICAM and IL-16.

A great body of evidence concerns the induction of inflammatory mediators, such as ICAM1 and IL16**,** in response to brain ischemic insult, that may contribute to the subsequent neuronal damage and death [19]–[23]; no data are available in SBI and LI patients. In animal models, the over-expression of ICAM1 has been related to enhanced leukocyte adherence and persistent activation in post-injury cerebral ischemia where its increase can contribute to stroke severity [19]–[21]. IL16 exerts a chemoattractant, pro-inflammatory and apoptotic activity contributing to a further worsening of ischemic damage in experimental and human brain ischemia [22], [23]. In humans, early accumulation of IL16 after cerebral infarction [23] has a role in the post-acute inflammatory response [22].

In SBI patients, the release of leukocytes adhesion molecules (ICAM1) is associated with the over-expression of proinflammatory cytokines (IL-18) and chemoattractants (MIG). IL18 is a reliable predictor of worse outcome in stroke [24] and myocardial infarction [25], [26]. MIG exerts angiostatic properties in experimental [27] and human coronary artery disease [23], [28]–[30]. At present, no data are available concerning its behavior in brain ischemic damage.

In animal models MIG and IL18 are also involved in atherosclerotic plaque progression and instability [30], [31].

According to Abramo et al [32]. MIG has been shown to be early expressed by IFN-γ stimulated mononuclear cells and to attract activated T-cells through the chemokine receptor CXCR3. Our panel did not include IFN-γ measurement and therefore the link between this proinflammatory cytokine and MIG could not be detected. However we cannot exclude that its increase may support the activation of downstream IFN-γ induced responses at least in SBI patients.

Conversely, some neuroprotective molecules and growth factors, namely SCF, have been suggested to intervene in limiting the extent of permanent ischemic damage after stroke [16], [33]. SCGFb has been demonstrated to exert angiogenic properties in animal [34] and human ischemic coronary artery syndrome [35]. No data are available concerning the role of SCF and SCGFb in SBI or LI. We could hypothesize that the increased SCF and SCGFb in SBI patients has a compensatory meaning, in the attempt to limit the extent of damage, favoring reparative mechanisms. In LI patients the detection of lower levels of both SCF and SCGFb might be related to less effective reparative mechanisms.

The peculiar immunological profiles detected in SBI and LI patients likely represent the expression of the low grade proinflammatory state accompanying the aging process, referred as inflammaging [36]. It can be hypothesized that quantitative and qualitative differences in the production of age-related inflammatory factors and reparative molecules could characterize the spectrum of brain ischemic events, including SBI, LI and major stroke.

An obvious reply to the above assertion is that in our study statistical differences in proinflammatory cytokines compared to younger healthy controls may reflect just immune alterations of the elderly towards a proinflammatory state. Moreover, several risk factors were significantly different among the patient groups, such as hypertension, diabetes mellitus, atrial fibrillation and carotid atheroma. In order to discriminate whether observed changes in SBI and LI such as ICAM or MIG are age-related or reflecting brain ischemic events age-matched controls without any vascular risk factors and ischemic events (excluded by radiology) in history are surely the best control group. We had, however, a great difficultly to find these subjects in our Neurologic clinic and therefore decided to include a younger healthy control group as a reference group for our cytokine level panel. We then adjusted our results for age and all vascular risk factor such as hypertension, diabetes mellitus, atrial fibrillation and carotid atheroma to better define the specific “inflammaging alterations” of SBI and LI. A further limitation of our study is the small patient sample size. A longitudinal assessment of cytokines, chemokines, and growth factors in a larger cohort of SBI and LI patients together with clinical and radiological findings (size,number and location of lesions) is therefore warranted.

### Conclusion

To our knowledge, this is the first study examining plasma levels of several immunological molecules in SBI and LI patients. Significant differences in peripheral inflammatory parameters emerged, suggesting the involvement of different immune-mediated mechanisms in these two conditions which are highly correlated and share classical vascular risk factors.

The inflammatory component in SBI and LI pathogenesis may have a significant role in influencing their progression and therefore the cerebrovascular burden.

## Supporting Information

Table S1 **List of the Ethics committees which approved the SILENCE study.** The table reports the complete list of Ethics committess that approved this study, including place, institutions and addresses.
(DOC)
