# Supplementary material for: Immunological Profile of Silent Brain Infarction and Lacunar Stroke
Source: PLoS One. 2013 Jul 9;8(7):e68428. doi: 10.1371/journal.pone.0068428 (PMC3706426; doi:10.1371/journal.pone.0068428)
Supplement: Table S1 — List of the Ethics committees which approved the SILENCE study. The table reports the complete list of Ethics committess that approved this study, including place, institutions and addresses. (DOC) [file pone.0068428.s001.doc]

**Supporting information**

**Table S1. List of the Ethics committees which approved the SILENCE study.**

| **Institution involved in the study** | **Ethical commitee** | **City** | **Address and contacts** |
| --- | --- | --- | --- |
| Universita’ degli studi di Perugia | COMITATO ETICO DELLE AZIENDE SANITARIE DELL`UMBRIA DI PERUGIA | Perugia | via della Rivoluzione, 16 - Ellera di Corciano (PG), 06073, PERUGIA, Telephone number: 0755170199, Fax: 0755182497, E-mail: [segreteria@ceasumbria.it](mailto:segreteria@ceasumbria.it), Website: www.ceasumbria.it |
| UNIVERSITA' DEGLI STUDI DI ROMA 'LA SAPIENZA' -(RM) | COMITATO ETICO DELL'AZIENDA POLICLINICO UMBERTO I DI ROMA | Rome | VIALE DEL POLICLINICO, 155, 00155, Rome, Telephone numbers: 064453395 – 0649970921, Fax: 064455345, E-mail: [comitato.etico@policlinicoumberto1.it](mailto:comitato.etico@policlinicoumberto1.it) |
| Marche Polytechnic University, Ospedali Riuniti Ancona | COMITATO ETICO DELL´AZIENDA OSPEDALIERO-UNIVERSITARIA OSPEDALI RIUNITI UMBERTO I - G.M. LANCISI - G. SALESI DI ANCONA | Ancona | VIA CONCA 71, 60126, Ancona, Telephone numbers: 0715965130 – 0715963667, Fax: 0715963106, E-mail: [d.amodio@ospedaliriuniti.marche.it, s.bolognini@ospedaliriuniti.marche.it](mailto:d.amodio@ospedaliriuniti.marche.it, s.bolognini@ospedaliriuniti.marche.it) |
| University of L’Aquila | COMITATO ETICO DELLA AUSL 4 DI L'AQUILA | L’Aquila, | Azienda U.S.L, 4 Comitato Etico ex P.O. Santa Maria di Collemaggio, 67100, L'Aquila, Telephone numbers: 0862368264 - Fax: 0862368264, E-mail: comitato.etico@asl-laquila.it |
